# A Unified Multiple-Target Positioning Framework for Intelligent Connected Vehicles

**DOI:** 10.3390/s19091967

**Published:** 2019-04-26

**Authors:** Zhongyang Xiao, Diange Yang, Fuxi Wen, Kun Jiang

**Affiliations:** 1 State Key Laboratory of Automotive Safety and Energy, School of Vehicle and Mobility, Tsinghua University, Beijing 100084, China; xiaozy15@mails.tsinghua.edu.cn (Z.X.); fuxi@chalmers.se (F.W.); jiangkun@mail.tsinghua.edu.cn (K.J.); 2 Department of Electrical Engineering, Chalmers University of Technology, SE-412 96 Gothenburg, Sweden

**Keywords:** vehicular localization, target positioning, high-definition map, vehicle-to-everything, intelligent and connected vehicles, intelligent transport system

## Abstract

Future intelligent transport systems depend on the accurate positioning of multiple targets in the road scene, including vehicles and all other moving or static elements. The existing self-positioning capability of individual vehicles remains insufficient. Also, bottlenecks in developing on-board perception systems stymie further improvements in the precision and integrity of positioning targets. Vehicle-to-everything (V2X) communication, which is fast becoming a standard component of intelligent and connected vehicles, renders new sources of information such as dynamically updated high-definition (HD) maps accessible. In this paper, we propose a unified theoretical framework for multiple-target positioning by fusing multi-source heterogeneous information from the on-board sensors and V2X technology of vehicles. Numerical and theoretical studies are conducted to evaluate the performance of the framework proposed. With a low-cost global navigation satellite system (GNSS) coupled with an initial navigation system (INS), on-board sensors, and a normally equipped HD map, the precision of multiple-target positioning attained can meet the requirements of high-level automated vehicles. Meanwhile, the integrity of target sensing is significantly improved by the sharing of sensor information and exploitation of map data. Furthermore, our framework is more adaptable to traffic scenarios when compared with state-of-the-art techniques.

## 1. Introduction

The intelligent transportation system (ITS) is one of the most indispensable components of the smart city concept that integrates sensing, control, information, and communication technologies into transportation [1]. In recent years, with the emergence of cutting-edge applications of ITS, the positioning of multiple targets, including vehicles and other elements has been playing an increasingly important role in improving safety, mobility, and efficiency [2,3,4]. For example, future intelligent connected vehicles (ICVs) require the positioning of their own real-time location with centimeter-level precision [5], and the awareness of all objects such as surrounding vehicles and vulnerable road users with significant integrity and confidence. In ITS, the positioning of vehicles and other targets are usually referred to as vehicular self-positioning and target localization, respectively. Although attention has been paid in these topics [6,7,8,9], there still exist many limitations that need to be eliminated.

### 1.1. Self-Positioning

Multiple self-positioning technologies are already present in the market, but none are effective under all road conditions and scenarios [10]. GNSS systems are widely employed in ITS devices, but they can support only low-precision navigation. Researchers have tried to integrate information from the base station and on-board sensors for error compensation. However, in dense urban environments where the signal is disturbed by surrounding buildings [5], even the most accurate GNSS with real-time kinematic correction and INS fusion schemes [11] cannot provide localization with adequate accuracy and stability.

Introducing new sources of information is an effective way of improving vehicular self-positioning. V2X communication, which has drawn increasing interest in recent years, renders information easily accessible to the vehicles connected [12,13,14]. The V2X-based (or cooperative) method aids in improving vehicular localization capability by employing the position information of other vehicles and relative measurements from their on-board sensors [15,16]. The integration of on-board sensors and V2X communication is shown to be more cost-effective than approaches based on high-quality sensors [17]. A general framework for multi-vehicular localization using pose graph optimization is proposed in [18], using vehicle-vehicle (V-V) measurements to improve the precision of vehicular localization. More recently, an implicit cooperative positioning algorithm that exploits the joint sensing of passive features is proposed in [19,20], and precludes the use of explicit V-V measurements. In addition to the use of ranging sensors, angle measurement-based cooperative localization is proposed in [21].

Maps are additional sources of information, and the locations of static elements can be used as references to improve the vehicular positioning capability [22]. In contrast to simultaneous localization and mapping (SLAM), in which a map is generated real-time [23], the map-based method assumes that maps are available in advance and aligns landmarks in the maps with on-board sensors to achieve independent positioning or as an aid to GNSS with INS system (GNSS/INS). As shown in Table 1, over the past few years, with the development of V2X, HD maps, which are characterized by high-accuracy and real-time updates, have grown to become standard and indispensable components of intelligent vehicles [4]. This also enables centimeter-level precision to be achieved in map-based localization [24].

The HD map-based method benefits from the high precision of the map used. For example, the digital map used in [25,26,27] is created from light detection and ranging (LiDAR) data and has a precision of up to 10 cm. A high-accuracy localization technique using urban environment maps for vehicles in motion is proposed in [28], and these maps are generated by integrating GNSS, LiDAR data, and on-board sensors. In addition, more features are used in the process of map alignment, which contributes to higher self-positioning precision. Traffic lights and visual lane markers are used as landmarks in [5,29], respectively. In a more recent study [26], a unified framework using more references in addition to the abovementioned features (lamp poles, traffic signs, etc.) is proposed, and self-localization with an accuracy of within 30 cm is achieved with merely a low-cost camera. While the abovementioned studies conduct map-based localization independently, there is much research that integrates it with other on-board sensors. A lane determination system that fuses on-board sensors, GNSS, and commercially available road network maps is proposed in [30]. A proof-of-concept study using INS and maps for vehicular localization in GNSS-denied environments is conducted in [31].

### 1.2. Target Localization

Apart from vehicular self-positioning, the relative localization of targets in the surrounding environment is another fundamental technology underpinning ICVs. This task is mainly undertaken by vehicle perception modules, and the positioning result is obtained in the vehicular coordinate instead of in the world coordinate. Although the recent decades have witnessed the rapid development of on-board sensors, the current on-board sensing technology still faces the following problems [4]. First, there is a trade-off between localization accuracy and cost. For example, low-cost cameras and radars can achieve accuracies of only several centimeters, while LiDAR systems with centimeter-level ranging accuracy are expensive [32]. Second, all sensors have limited sensing ranges, and the occlusion of sensors by other vehicles and objects is a frequent occurrence [33]. Irrespective of the number of sensors equipped in the vehicle, the perception of the environment remains incomplete. Attempts to improve the perception of bicycles have already encountered bottlenecks to a certain extent.

Studies using the perceptual information of other vehicles to improve the integrity of target localization have proven to be effective. This is because other vehicles in the network may have seen a target that cannot be seen by the ego-vehicle because of occlusion or limited field of view. A vehicle-to-vehicle (V2V) communication and map merging-based cooperative perception system to extend the perception range beyond line of sight and field of view is proposed in [34]. In [35], the results of the awareness of other vehicles are integrated into the ego-vehicle’s perception system as virtual sensors to achieve perception enhancement. In [36], a multi-vehicle perception framework combining image and semantic features is proposed, and experiments have proved that the problem of front-vehicle occlusion can be solved. In these studies, the problems of self-positioning and localization of other targets were considered separately, which rendered the effect of the fusion very sensitive to their relative positioning. In addition, these articles do not provide quantitative analyses of the integrity of the results perceived.

Maps also contribute to the relative positioning of targets. For example, the geometry of intersection can be directly extracted from a HD map for motion planning and control [37]. This reduces the pressure on the vehicle-mounted sensing system, but relies on vehicular self-positioning. Incorrect self-positioning greatly affects decision-making. Other works integrate the semantic and geometric prior knowledge in HD maps with the on-board sensing system to improve positioning confidence. In [38,39], a prior probability map is generated in a bird’s-eye view or image plane to aid understanding of the scene. Recently, a neural network incorporating prior knowledge with on-board sensors was presented in [40]. However, these studies treat the map only as an auxiliary tool for perception to improve the recognition result without improving the integrity of perception. Moreover, these studies also rely on the accuracy of vehicular positioning.

### 1.3. Contributions

In general, recent research exploits additional sources of information to improve vehicular self-positioning and localization of other targets. However, to the best of our knowledge, V2X and HD maps are considered separately in the literature, while the positioning of the vehicles themselves and those of other targets are usually treated as different modules. In this paper, we propose a unified theoretical positioning framework for multiple targets for ICVs. The bottlenecks of vehicular self-positioning and target localization can be eliminated. Our main contributions are summarized as follows.

- A unified theoretical framework for vehicular self-positioning and relative localization of targets based on V2X is proposed, and it can integrate data from the on-board sensors in the vehicular network and HD maps with GNSS/INS measurements into a unified system.
- By cooperative positioning, accuracy of under 0.2 m can be achieved in terms of self-positioning and relative localization of targets in urban areas using low-cost GNSS/INS, on-board sensors, and widely equipped HD maps. Simultaneously, the target sensing range is extended beyond the line of sight and field of view, and this greatly improves the integrity of perception.
- Furthermore, compared with state-of-the-art techniques, the proposed framework places fewer demands on vehicular network nodes’ density and the amount of vehicle-to-target measurements.

The remainder of the paper is organized as follows. In Section 2, the system model is provided. The development of the proposed joint multiple-target positioning for ICVs is detailed in Section 3. Detailed implementation aspects are introduced in Section 4. Theoretical studies are explained in Section 5. Numerical results are given in Section 6. Finally, we conclude the paper in Section 7.

## 2. Problem Formulation

Firstly, we describe the targets in a traffic scene in this section.

- Targets: All objects related to vehicle driving, including the connected vehicles themselves and the elements that constitute the environment.
- Connected vehicles: Vehicles in the vehicular network that can obtain information from other vehicles and HD maps.
- Features: Static targets that can be associated with HD maps, e.g., lamps, trees, traffic lights, and traffic signs.
- Objects: Targets, both static and moving, that do not exist in HD maps. These can be pedestrians, bicycles, and disconnected vehicles, all of which are unlabeled on the map.

**Figure 1:**
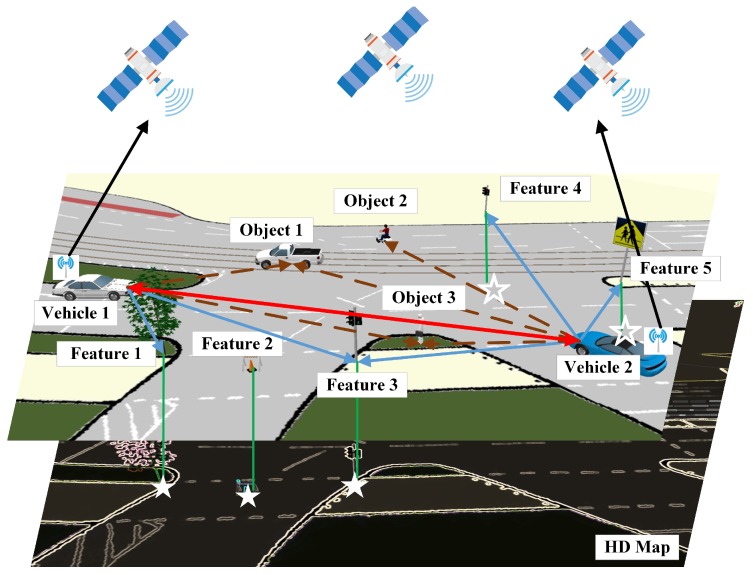
A demonstration of the multiple targets positioning scenario.

Consider a vehicular network scenario with a set of $N_{v}$ interconnected vehicles $V=\left\{1,2,\cdots ,N_{v}\right\}$, as shown in Figure 1. At time *t*, let $x_{i,t}^{\left(V\right)}$ be the position and orientation of connected vehicle *i* in the global coordinate (see Equation (1)).

(1) $$x_{i,t}^{\left(V\right)}=\left[p_{i,t}^{\left(V\right)},\theta_{i,t}^{\left(V\right)}\right]={\left[x_{i,t}^{\left(V\right)},y_{i,t}^{\left(V\right)},\theta_{i,t}^{\left(V\right)}\right]}^{T},i\in V$$

A set of $N_{f}$ static features $F=\left\{1,2,\cdots ,N_{f}\right\}$ also exists in the scene. Their positions are stored on a HD map with noise and, although not necessary, can be captured by the on-board sensors. We use Equation (2) to describe the two-dimensional position of the *j*th feature.

(2) $$x_{j,t}^{\left(F\right)}=p_{j,t}^{\left(F\right)}={\left[x_{j,t}^{\left(F\right)},y_{j,t}^{\left(F\right)}\right]}^{T},j\in F$$

In addition, we also consider a set of $N_{o}$ objects, moving or static, $O=\left\{1,2,\cdots ,N_{o}\right\}$, which is described in Equation (3).

(3) $$x_{k,t}^{\left(O\right)}=p_{k,t}^{\left(O\right)}={\left[x_{k,t}^{\left(O\right)},y_{k,t}^{\left(O\right)}\right]}^{T},k\in O$$

It must be noted that we do not estimate the orientations of the features and objects because the planning module usually does not require this information.

Our task is to estimate the localization and orientation of all the connected vehicles,

(4) $$X_{t}^{\left(V\right)}=\left[x_{1,t}^{\left(V\right)}\;\cdots \;x_{N_{v},t}^{\left(V\right)}\right],$$

We also attempt to localize the features and objects (see Equation (5))

(5) $$\begin{array}{cc} X_{t}^{\left(F\right)} & =\left[x_{1,t}^{\left(F\right)}\;\cdots \;x_{N_{f},t}^{\left(F\right)}\right]\\ X_{t}^{\left(O\right)} & =\left[x_{1,t}^{\left(O\right)}\;\cdots \;x_{N_{o},t}^{\left(O\right)}\right] \end{array}$$

Based on Equations (4) and (5), we can obtain the relative localization of other targets by transforming their location into the vehicles’ coordinate system.

In terms of the measurements, a target may be captured by a vehicle if it is within the vehicle’s sensing range and without any occlusions. As shown in Figure 1, the target can be a connected vehicle, a feature, or an object, which are indicated with red, blue, and brown arrows, respectively. Its measurement model is described as Equation (6).

(6) $$z_{i,j,t}^{\left(\Xi \right)}=h^{\left(S\right)}\left(p_{j,t}^{\left(\Xi \right)},x_{i,t}^{\left(V\right)}\right)+v_{i,j,t}^{\left(S\right)}$$

where $\Xi \in \left\{V,F,O\right\}$ and $v_{k,j,t}^{\left(S\right)}∼N\left(0,R_{k,j,t}^{\left(S\right)}\right)$ is additive white Gaussian measurement noise with covariance $R_{k,j,t}^{\left(S\right)}$, $h^{\left(S\right)}\left(p_{j,t}^{\left(\Xi \right)},x_{k,t}^{\left(V\right)}\right)$ is a function which denotes the measurement of target at position $p_{j,t}^{\left(\Xi \right)}$ from vehicle $x_{k,t}^{\left(V\right)}$.

The connected vehicles are also equipped with GNSS/INS, which can provide measurements of their localization and orientation. The corresponding measurements are indicated with black arrows in Figure 1. Similarly, we treat the map as a virtual sensor with measurements pertaining to an associated feature as shown with green arrows. The measurement of GNSS/INS on vehicle *i* and that of HD map on feature *j* is indicated by Equations (7) and (8).

(7) $$z_{i,t}^{\left(G\right)}=h^{\left(G\right)}\left(p_{i,t}^{\left(V\right)}\right)+v_{i,t}^{\left(G\right)}$$

and

(8) $$z_{j,t}^{\left(M\right)}=h^{\left(M\right)}\left(p_{j,t}^{\left(F\right)}\right)+v_{j,t}^{\left(M\right)}$$

where $v_{i,t}^{\left(G\right)}∼N\left(0,R_{i,t}^{\left(G\right)}\right)$ is the measurement noise of GNSS/INS, and $v_{j,t}^{\left(M\right)}∼N\left(0,R_{j,t}^{\left(M\right)}\right)$ denotes the measurement noise from the map.

If we consider this problem as analogous to a distributed sensor network, the features and connected vehicles are static and mobile anchors, respectively, and their locations are constrained by the HD map and GNSS/INS. The objects are static or mobile nodes, and the on-board sensors generate constraints between the vehicle and nodes. For the vehicles, additional constraints arise from the V-V measurements.

## 3. The Unified Multiple-Target Positioning Framework

The objective of multiple-target positioning in this paper is to estimate the states $X_{t}=\left[X_{t}^{\left(V\right)},X_{t}^{\left(F\right)},X_{t}^{\left(O\right)}\right]$, from measurements,

(9) $$Z_{t}=\left[z_{k,j,t}^{\left(S\right)},z_{k,t}^{\left(G\right)},z_{l,t}^{\left(M\right)}\right],$$

where k∈V,j∈{V,F,O},l∈F. From a probabilistic perspective, the maximum likelihood estimation of $X_{t}$ is given by Equation (10).

(10) $$X_{t}^{*}=\arg\max P\left(Z_{t}|X_{t}\right)$$

where $P\left(Z_{t}|X_{t}\right)$ is the likelihood of the measurements $Z_{t}$ given the states $X_{t}$. The conditional distribution of the on-board sensor measurements in Equation (6) is given by Equation (11), where $P\left(z_{k,j,t}^{\left(S\right)}|p_{j,t}^{\left(\Xi \right)},x_{k,t}^{\left(V\right)}\right)$ denotes the probability distribution of measurement $z_{k,j,t}^{\left(S\right)}$ given the states $p_{j,t}^{\left(\Xi \right)}$ and $x_{k,t}^{\left(V\right)}$. $N\left(h^{\left(S\right)}\left(p_{j,t}^{\left(\Xi \right)},x_{k,t}^{\left(V\right)}\right),R_{k,j,t}^{\left(S\right)}\right)$ denotes a normal distribution with expectation $h^{\left(S\right)}$ and variance $R_{k,j,t}^{\left(S\right)}$.

(11) $$P\left(z_{k,j,t}^{\left(S\right)}|p_{j,t}^{\left(\Xi \right)},x_{\left(k,t\right)}^{\left(V\right)}\right)=N\left(h^{\left(S\right)}\left(p_{j,t}^{\left(\Xi \right)},x_{k,t}^{\left(V\right)}\right),R_{k,j,t}^{\left(S\right)}\right)$$

Similarly, we get the conditional distributions of the measurement from GNSS/INS $P\left(z_{i,t}^{\left(G\right)}|p_{i,t}^{\left(V\right)}\right)$ and map $P\left(z_{j,t}^{\left(M\right)}|p_{j,t}^{\left(F\right)}\right)$ given states of vehicles $p_{i,t}^{\left(V\right)}$ or feature states $p_{j,t}^{\left(F\right)}$ (see Equations (12) and (13)). Their distributions are also normal with perception model *h* as expectation.

(12) $$P\left(z_{i,t}^{\left(G\right)}|p_{i,t}^{\left(V\right)}\right)=N\left(h^{\left(G\right)}\left(p_{i,t}^{\left(V\right)}\right),R_{i,t}^{\left(G\right)}\right)$$

and

(13) $$P\left(z_{j,t}^{\left(M\right)}|p_{j,t}^{\left(F\right)}\right)=N\left(h^{\left(M\right)}\left(p_{j,t}^{\left(F\right)}\right),R_{j,t}^{\left(M\right)}\right)$$

**Remark** **1.** *Given a set of independent and identically distributed (i.i.d.) data $D=\{x_{n},n=1,2,\cdots ,N\}$, where observation $x_{n}\in R^{D\times 1}$ is drawn from a multivariate Gaussian distribution $N(x_{n};\mu_{n},R_{n})$. The log-likelihood of the data set can be written as Equation (14).*

(14) $$L\left(D\right)=-\frac{1}{2}\sum_{n=1}^{N}\left(\ln\left({\left(2\pi \right)}^{D}\det\left(R_{n}\right)\right)+{e_{n}}^{T}R_{n}^{-1}e_{n}\right)$$

*where $e_{n}=x_{n}-\mu_{n}$.*

According to Remark 1, the maximization of L(D) is equivalent to the minimization of J(D) (see Equation (15)). The problem is solved with optimization as described in Section 4.

(15) $$J\left(D\right)=\sum_{n=1}^{N}e_{n}^{T}{R_{n}}^{-1}e_{n}$$

Considering (11)–(13) and assuming that the three types of measurements are independent, the joint probability density can be factorized as given in Equation (16).

(16) $$\begin{array}{cc} P\left(Z_{t}|X_{t}\right)= & P\left(Z_{t}^{\left(V\right)},Z_{t}^{\left(F\right)},Z_{t}^{\left(O\right)}|X_{t}\right)\\ = & \prod_{k,j}P\left(z_{k,j,t}^{\left(S\right)}|p_{j,t}^{\left(\Xi \right)},x_{\left(k,t\right)}^{\left(V\right)}\right)\\ & \prod_{i}P\left(z_{i,t}^{\left(G\right)}|p_{i,t}^{\left(V\right)}\right)\prod_{j}P\left(z_{j,t}^{\left(M\right)}|p_{j,t}^{\left(F\right)}\right) \end{array}$$

The maximization of $P(Z_{t}|X_{t})$ can be reformulated as the following nonlinear least squares problem (see Equation (17)).

(17) $$\begin{array}{cc} X_{t}^{*}=\arg\min & \sum_{k}\sum_{j}{\left(e_{k,j,t}^{\left(S\right)}\right)}^{T}{\left(R_{k,j,t}^{\left(S\right)}\right)}^{-1}e_{k,j,t}^{\left(S\right)}\\ & +\sum_{i}{\left(e_{i,t}^{\left(G\right)}\right)}^{T}{\left(R_{i,t}^{\left(G\right)}\right)}^{-1}e_{i,t}^{\left(G\right)}\\ & +\sum_{j}{\left(e_{j,t}^{\left(M\right)}\right)}^{T}{\left(R_{j,t}^{\left(M\right)}\right)}^{-1}e_{j,t}^{\left(M\right)} \end{array}$$

To enable insightful visualization, the nonlinear least–squares problem is interpreted in terms of inference over a factor graph [41]. This graph consists of 2 types of nodes: variable nodes, which represent the state $X_{t}$, and factor nodes, which represent the constraints to on the variables. The factor nodes can be further divided into bi-directed nodes, which denote the constraints for 2 states (from the on-board sensor measurements), and directed prior nodes, which denote the constraints from the map and GNSS/INS.

**Figure 2:**
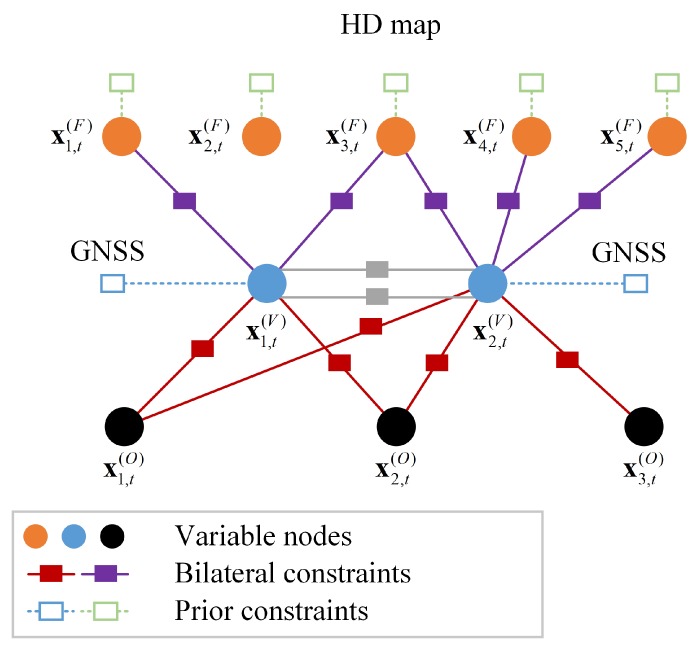
The proposed framework interpreted as inference on factor graphs.

As shown in Figure 2, for each measurement, we have the following factors.

- Factor between the variables V and Ξ={V,F,O}, on behalf of the constraints of V-V, vehicle-feature (V-F), vehicle-object (V-O), as expressed in Equation (18).
  (18) $$\phi_{k,j,t}=P\left(z_{k,j,t}^{\left(S\right)}|p_{j,t}^{\left(\Xi \right)},x_{\left(k,t\right)}^{\left(V\right)}\right)$$
- Factor between the variables V and GNSS/INS, on behalf of the constraints from GNSS/INS, as expressed in Equation (19).
  (19) $$\phi_{i,t}=P\left(z_{i,t}^{\left(G\right)}|p_{i,t}^{\left(V\right)}\right)$$
- Factor between the variables F and the map, on behalf of the constraints from the HD map, as expressed in Equation (20).
  (20) $$\phi_{j,t}=P\left(z_{j,t}^{\left(M\right)}|p_{j,t}^{\left(F\right)}\right)$$

The joint probability in Equation (16) can then be rewritten as the product of all the factors.

(21) $$P\left(Z_{t}|X_{t}\right)=\prod_{k,j,t}\phi_{k,j,t}\prod_{i,t}\phi_{i,t}\prod_{j,t}\phi_{j,t}$$

We can clearly see the constraints applied on each node in the factor graph.

## 4. Implementation Aspects

In this section, we introduce the implementation aspects related to the hypothesis on vehicle perception, the measurement model, optimization, and data association.

### 4.1. Perception Demands and Sensing Capability of Vehicles

We assume that due to occlusion and the limitations of perception range, the vehicle cannot completely locate the desired target. In this section, we explain the hypothesis of this work. It should be noted that our hypothesis is based on typical perceptual systems, but can be easily adapted to other forms.

**Figure 3:**
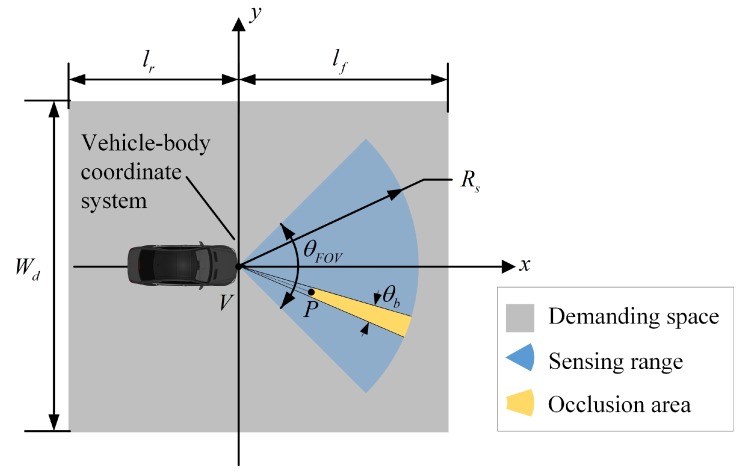
Sensing range and perception demand.

As shown in Figure 3, we identify the scope of targets that need to be localized by a vehicle as “demanding space” and assume that it is a rectangle that can be quantitatively described by $l_{f}$ and $l_{r}$, i.e., the distances that the vehicle requires to sense ahead of and behind itself, respectively, and $W_{d}$, the range that should be perceived laterally. We assume that the vehicle sensing range is a forward-facing cone with a radius of $R_{s}$, and the field of view is $\theta_{FOV}$.

To consider situations of occlusion, we assume that there is an object *P* in the sensing range, and that the area outside *P* in the sector with line VP as the axis of symmetry is regarded as the occlusion area. Thus, only the blue area in Figure 3 can be perceived. The limitations in sensing range and occlusion constitute the blind spots of environment perception.

### 4.2. Measurement Model

In this work, we assume that data from the on-board sensors are in a 2D vehicular coordinate fashion where $h^{\left(S\right)}∼R^{2\times 1}$, correspond to the measurements from low-cost cameras. This can be easily adapted to other measurement types, such as polar coordinates in LiDAR measurements. The measurement model is expressed as,

(22) $$h^{\left(S\right)}\left(p_{j,t}^{\left(\Xi \right)},x_{i,t}^{\left(V\right)}\right)=R^{-1}\left(\theta_{i,t}^{\left(V\right)}\right)\left[p_{j,t}^{\left(\Xi \right)}-p_{i,t}^{\left(V\right)}\right],$$

where the rotation matrix is expressed as,

(23) $$R\left(\theta_{i,t}^{\left(V\right)}\right)=\left[\begin{array}{cc} \cos\left(\theta_{i,t}^{\left(V\right)}\right) & -\sin\left(\theta_{i,t}^{\left(V\right)}\right)\\ \sin\left(\theta_{i,t}^{\left(V\right)}\right) & \cos\left(\theta_{i,t}^{\left(V\right)}\right) \end{array}\right],$$

and the covariance of the on-board sensors’ measurements is given in Equation (24).

(24) $$R_{k,j,t}^{\left(S\right)}=\left[\begin{array}{cc} \delta_{sensor}^{2} & 0\\ 0 & \delta_{sensor}^{2} \end{array}\right]$$

We assume that the GNSS/INS can provide the measurements of the coordinates and angles of the vehicles. There are many studies on modeling the measurements noise of GNSS/INS [42,43]. In this paper, we simplify the error of GNSS to Gaussian distribution, and the measurement model and uncertainty of the GNSS/INS on vehicle *i* are indicated as Equations (25) and (26). Our framework is also suitable for extending to other error assumptions.

(25) $$h^{\left(G\right)}\left(p_{i,t}^{\left(V\right)}\right)=p_{i,t}^{\left(V\right)}$$

and

(26) $$R_{i,t}^{\left(G\right)}=\left[\begin{array}{ccc} \delta_{\mathrm{GNSS},l}^{2} & 0 & 0\\ 0 & \delta_{\mathrm{GNSS},l}^{2} & 0\\ 0 & 0 & \delta_{\mathrm{GNSS},\theta }^{2} \end{array}\right]$$

In commercial HD maps, the coordinates of the features are provided along with noise, so we formulate the measurement mode and covariance matrix as expressed by Equations (27) and (28).

(27) $$h^{\left(M\right)}\left(p_{j,t}^{\left(F\right)}\right)=p_{j,t}^{\left(F\right)}$$

(28) $$R_{j,t}^{\left(M\right)}=\left[\begin{array}{cc} \delta_{map}^{2} & 0\\ 0 & \delta_{map}^{2} \end{array}\right]$$

### 4.3. Optimized Variable Allocation and Data Association

The optimized variables are allocated to observations within the demanding space of perception. Unlike in a traditional multi-vehicle cooperative system, barring objects and features captured by vehicles, features that are not seen by any vehicle but are within the demanding space are also included in the optimized variables, and further optimized to yield the results of perception.

Observations that are associated are merged into existing variables and form constraints in the process of optimization. There are many methods that can be applied to our framework [44,45]. In this study, we assume that the vehicle’s on-board sensors and HD maps can provide enough semantic clue to identify objects. The association algorithm itself is beyond the scope of this article.

### 4.4. Optimization Problem Solving

In this study, the nonlinear optimization problem in Equation (17) is solved via the Levenberg-Marquardt method [46]. We reorganize the residuals of time *t* into one vector, as expressed in Equation (29).

(29) $$e={\left(\;e_{ij}^{\left(S\right)}\;e_{kp}^{\left(S\right)}\;e_{m}^{\left(G\right)}\;e_{q}^{\left(M\right)}\;\right)}^{T}$$

where $e_{ij}^{\left(S\right)}$ is the residual of measurement from the on-board sensor of vehicle *i* to vehicle *j*. $e_{kp}^{\left(S\right)}$ is the residual of measurement from the on-board sensor of vehicle *k* to feature or object *p*. $e_{m}^{\left(G\right)}$ is the residual of GNSS/INS measurement of vehicle *m*, and $e_{q}^{\left(M\right)}$ is the residual of measurement from HD map to feature *q*. The optimized variable at time *t* can then be rewritten as,

(30) $$\begin{array}{c} X=\left(x_{i}^{\left(V\right)}\;x_{j}^{\left(V\right)}\;x_{k}^{\left(V\right)}\;x_{p}^{\left(\Xi \right)}\;x_{m}^{\left(V\right)}\;x_{q}^{\left(F\right)}\right), \end{array}$$

where $\Xi \in \left\{F,O\right\}$. Let R be the overall covariance matrix such that

(31) $$R=diag\left(R^{\left(S\right)}\;R^{\left(S\right)}\;R^{\left(G\right)}\;R^{\left(M\right)}\right).$$

The cost function can be rewritten as,

(32) $$f\left(X\right)={\left(R^{-\frac{1}{2}}e\right)}^{T}(R^{-\frac{1}{2}}e).$$

We can get the Jacobian matrix (see Equation (33)).

(33) $$\begin{array}{c} J\left(X\right)=\frac{\partial (R^{-\frac{1}{2}}e)}{\partial X}=\\ R^{-\frac{1}{2}}\left[\begin{array}{cccccc} \frac{\partial e_{ij}^{\left(S\right)}}{\partial x_{i}^{\left(V\right)}} & \frac{\partial e_{ij}^{\left(S\right)}}{\partial x_{j}^{\left(V\right)}} & 0 & 0 & 0 & 0\\ 0 & 0 & \frac{\partial e_{kp}^{\left(S\right)}}{\partial x_{k}^{\left(V\right)}} & \frac{\partial e_{kp}^{\left(S\right)}}{\partial x_{p}^{\left(\Xi \right)}} & 0 & 0\\ 0 & 0 & 0 & 0 & \frac{\partial e_{m}^{\left(G\right)}}{\partial x_{m}^{\left(V\right)}} & 0\\ 0 & 0 & 0 & 0 & 0 & \frac{\partial e_{q}^{\left(M\right)}}{\partial x_{q}^{\left(F\right)}} \end{array}\right] \end{array}$$

The initial values of the optimization iterations are given as follows. The vehicular position and attitude are calculated by the measurements of the GNSS/INS. The positions of features are determined by the map, and the initial positions of objects are calculated by converting the positions measured by the on-board sensors to the geodetic coordinate system according to the initial vehicle position and attitude. The cost function (17) can be minimized towards zero by iterations:

(34) $$X_{k+1}\leftarrow X_{k}-{\left(J^{T}J+\lambda diag\left(J^{T}J\right)\right)}^{-1}J^{T}f\left(X_{k+1}\right)$$

where λ is determined by the Levenberg-Marquardt method, and J and f are defined in Equations (32) and (33), respectively.

## 5. Theoretical Analysis on the Framework Performance

The proposed multiple-target positioning framework aims to solve a parameter estimation problem. Its performance can be evaluated either numerically or theoretically. In this section, the lower bounds on the estimation errors are determined from theoretical studies. As one of the most widely used lower bounds, the Cramér-Rao lower bound (CRLB) is chosen as the performance benchmark. The framework is performance-bound in terms of the minimum achievable variance provided by any unbiased estimators.

Assume that a deterministic signal $s_{t}\left(\theta \right)$ with an unknown vector parameter θ is observed in white Gaussian noise as Equation (35).

(35) $$z_{t}=h_{t}\left(\theta \right)+v_{t}$$

where $v_{t}∼N\left(0,C_{t}\right)$. We wish to estimate θ from z. The Fisher information matrix [47] is given by Equation (36).

(36) $${\left[I(\theta )\right]}_{m,n}={\left[\frac{\partial h_{t}\left(\theta \right)}{\partial \theta_{m}}\right]}^{T}C_{t}^{-1}\left[\frac{\partial h_{t}\left(\theta \right)}{\partial \theta_{n}}\right]$$

Taking the inverse of I(θ), the CRLB for the parameters is then obtained from its diagonal elements. The CRLB for $\theta_{m}$ is the (m,m) entry of $I^{-1}\left(\theta \right)$.

For the proposed framework, the following measurements are considered.

$z_{i,t}^{\left(V\right)}$ vehicle i∈V, measured from GNSS/INS;
$z_{il,t}^{\left(V2V\right)}$ measured from vehicle *i* to vehicle *l*, where i∈Vandl∈V;
$z_{j,t}^{\left(F\right)}$ feature j∈F, measured from the HD map;
$z_{ij,t}^{\left(V2F\right)}$ vehicle i∈V to feature j∈F, measured from the vehicle’s on-board sensors; and
$z_{ik,t}^{\left(V2O\right)}$ vehicle *i* to object k∈O.

For convenience, all the measurements available are reformulated to the following compact form:

(37) $$z_{t}=h_{t}\left(\theta \right)+v_{t}=\left[\begin{array}{c} z_{i,t}^{G}\\ z_{j,t}^{M}\\ z_{i,j,t}^{\Xi } \end{array}\right]+v_{t}$$

where $z_{i,t}^{G}$, $z_{j,t}^{M}$ and $z_{i,j,t}^{\Xi }$ are defined in Equations (6)–(8), respectively. The unknown parameters are obtained from Equation (38).

(38) $$\theta^{T}=\underset{i\in V}{\underset{︸}{[x_{i,t}^{\left(V\right)}\;y_{i,t}^{\left(V\right)}\;\theta_{i,t}}}\;\underset{j\in F}{\underset{︸}{x_{j,t}^{\left(F\right)}\;y_{j,t}^{\left(F\right)}}}\;\underset{k\in O}{\underset{︸}{x_{k,t}^{\left(O\right)}\;y_{k,t}^{\left(O\right)}]}}$$

We observe that $z_{t}$ is Gaussian distributed with mean $h_{t}\left(\theta \right)$ and covariance matrix $C_{t}$:

(39) $$z_{t}∼N\left(h_{t}\left(\theta \right),C_{t}\right)$$

The CRLB for θ is obtained by substituting $h_{t}\left(\theta \right)$ and $C_{t}$ into Equation (36).

## 6. Numerical Results

In this section, we discuss the simulation experiments conducted under typical vehicular network scenarios to verify the localization and perception capacity results of the proposed algorithm. We also demonstrate its environmental adaptability in subsequent discussions on factors that influence the final performance by considering different scene configurations.

**Figure 4:**
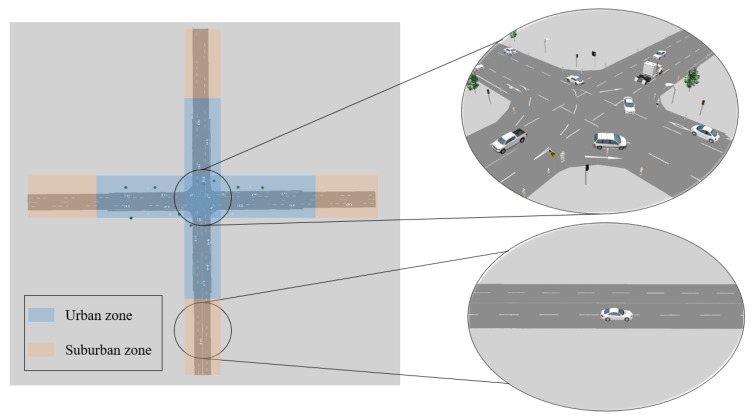
The considered intersection simulated in VISSIM.

As shown in Figure 4, we build an intersection with 2 two-way two-lane roads. This scenario consists of a busy urban area and a suburban area. The trajectories of all the vehicles and objects as well as the traffic scene configuration come from VISSIM, a behavior-based traffic flow simulator [48]. Each road is 330 m long. In the middle, until approximately 200 m from the intersection, we simulate a busy urban scenario with lamps, traffic lights, and traffic signs located randomly on the roadside. Pedestrians walking around the road and across the intersection are generated. Outside of and far from the intersection, nothing is placed on the roadside, which simulates the scenario of a suburban area. In the simulation, connected and disconnected vehicles start from one end of the road simultaneously, then travel straight or turn left or right at the intersection, and exit the scene almost simultaneously. Therefore, vehicles are in the urban area in the middle section of the simulation steps, and the starting and ending segments correspond to suburban scenes.

### 6.1. Performance in a Typical Scenario

First, we validate the effectiveness of our algorithm in a fixed scenario and compare it with the method proposed by Gloria et al. [19], as well as the theoretical bound CRLB. We set the accuracy of each measurement to that of low-cost devices. The configuration for the scenario and each measurement are as follows.

- $N_{v}=6$
- $N_{f}=23$ (15 lamps, 4 traffic lights, and 4 traffic signs)
- $N_{o}=18$ (10 pedestrians, and 8 disconnected vehicles)
- $\delta_{sensor}=$ 0.25 m, and $\delta_{map}=$ 0.05 m
- $\delta_{\mathrm{GNSS},l}=$ 2.5 m, and $\delta_{\mathrm{GNSS},\theta }=$ 0.1 rad
- $\theta_{\mathrm{FOV}}=$ 70 m, $R_{s}=$ 80 m, and $\theta_{b}=2^{\circ }$
- $L_{f}=$ 100 m, $L_{r}=$ 30 m, and $W_{d}=$ 60 m

**Figure 5:**
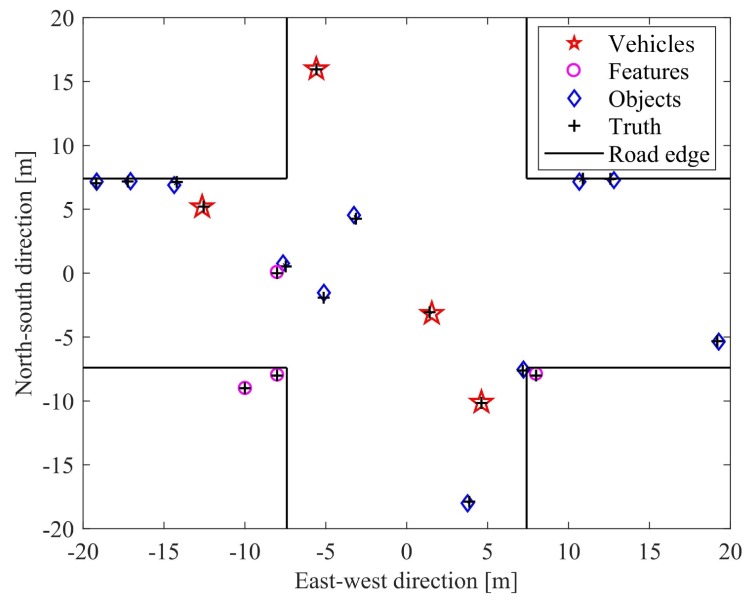
Multiple-target position performance in the urban area.

We run the simulation 200 times, and noise is added to the measurements independently for each iteration. One localization result of the vehicles, objects, and features, and their true positions in the urban area is shown in Figure 5. As the 6 vehicles face similar scenes in every simulation step, we statistically analyze the positioning errors of all the vehicles. The root-mean-square error (RMSE) of self-positioning for all 6 vehicles at simulation time *t* is calculated using Equation (40).

(40) $${\mathrm{RMSE}}_{t}^{\left(V\right)}=\sqrt{\frac{1}{MN}\sum_{j=1}^{N}\sum_{i=1}^{M}{∥\begin{array}{c} {\hat{p}}_{i,j,t}^{\left(V\right)}-p_{i,j,t}^{\left(V\right)} \end{array}∥}_{2}^{2}}$$

where ${\hat{p}}_{i,j,t}^{\left(V\right)}$ is the self-positioning result of vehicle *i* at the *j*th run at simulation step *t*, and $p_{i,j,t}^{\left(V\right)}$ is its corresponding ground truth. The self-positioning mean-square error MSE bound is calculated using Equation (41)

(41) $${\mathrm{CRLB}}_{t}=\frac{1}{M}\sum_{i=1}^{M}\left(\mathrm{CRLB}\left(x_{i,t}^{\left(V\right)}\right)+\mathrm{CRLB}\left(y_{i,t}^{\left(V\right)}\right)\right)$$

where $\mathrm{CRLB}(x_{i,t}^{\left(V\right)})$ and $\mathrm{CRLB}(y_{i,t}^{\left(V\right)})$ are the CRLBs of the *x* and *y* coordinates of vehicle *i* at simulation step *t*.

**Figure 6:**
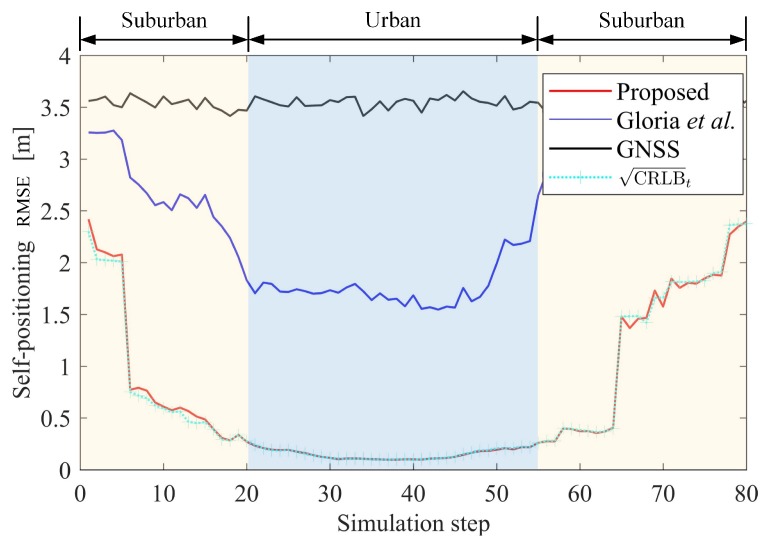
Self-localization error.

As shown in Figure 6, the proposed method is compared with that of Gloria et al. [19], as well as the RMSE bound ${\sqrt{\mathrm{CRLB}}}_{t}$ (see Equation (41)).

It is obvious that compared with the original GNSS measurements, we obtain a significantly improved positioning result by using the information from V2X and the HD map. In particular, in the urban area (simulation steps 21–55), our algorithm achieves high positioning accuracy (0.16 m), which is also lower than that of the method in [19] (1.79 m). Our positioning accuracy is close to the theoretical lower bound given by CRLB, which shows that we have effectively used all valuable information.

**Figure 7:**
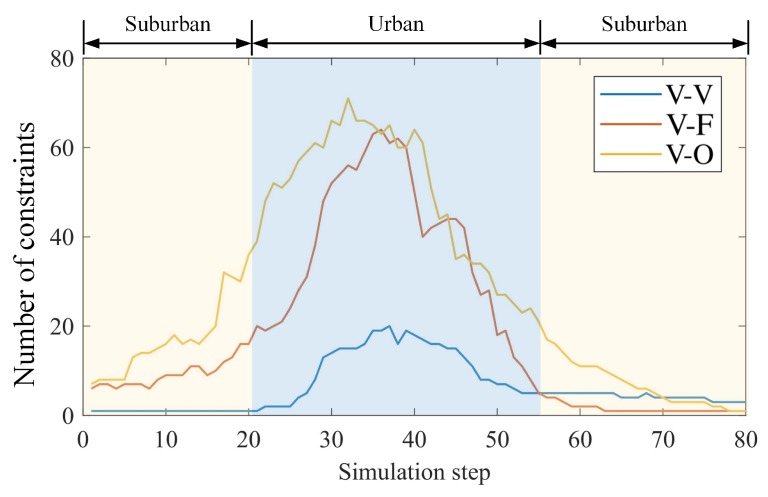
Amount of constraints used at every simulation step.

We also give the number of constraints at each simulation time in Figure 7. Overall, the greater the number of constraints available, the better our positioning results are. In fact, the study in [19] only uses GNSS and V-O constraints, while we have used additional constraints including V-F, V-V, and prior constraints of the HD map.

In the suburban area where the sensing ranges of different vehicles have little overlap, the method proposed in [19] acts ineffectively as few constraints are available. However, our positioning is still a significant improvement in these challenging areas. Environmental adaptability will be further discussed in the next section.

**Figure 8:**
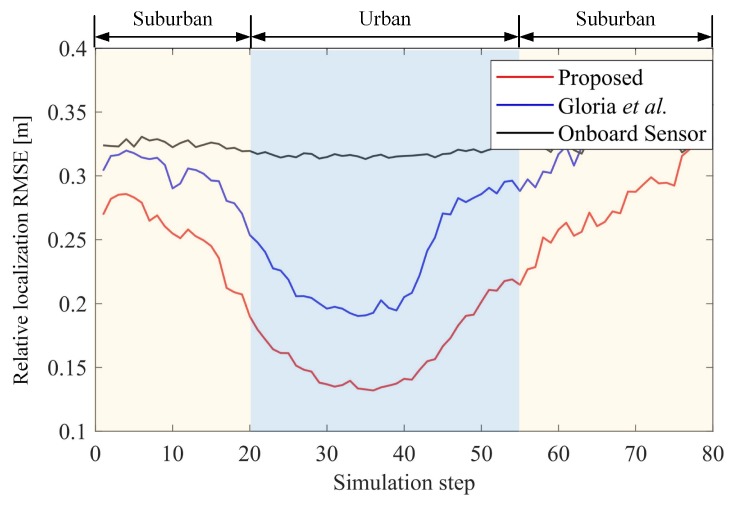
Relative positioning error of surrounding targets.

In terms of the positioning of other targets, we compared both the target location precision and sensing integrity. We transform the positioning results of these targets into the body coordinate system (i.e., the vehicle coordinate system shown in Figure 3 for analysis, as this analysis is consistent with the vehicle sensing system. The target positioning accuracy of a vehicle is evaluated by the RMSE of all targets within the demanding space (see Equation (42)).

(42) $${\mathrm{RMSE}}_{i,t}^{\left(T\right)}=\sqrt{\frac{1}{ON}\sum_{k=1}^{O}\sum_{j=1}^{N}{∥\begin{array}{c} {\hat{p}}_{k,j,t}^{\left(T\right)}-p_{k,j,t}^{\left(T\right)} \end{array}∥}_{2}^{2}}$$

where ${\hat{p}}_{k,j,t}^{\left(T\right)}$ is the localization result of the *k*th target in the vehicle coordinate system at the *j*th runtime, and $p_{k,j,t}^{\left(T\right)}$ is its truth position. The RMSE of relative localization i.e., ${\mathrm{RMSE}}_{t}^{\left(T\right)}$ is defined as the root mean square of the location of all vehicles. In fact, such a result is affected by both the absolute positioning and vehicular self-positioning, which makes our analysis more rigorous.The result is shown in Figure 8. In the urban area, the RMSE is 0.17 m; it is much smaller than that (0.24 m) obtained by Gloria’s method as well as that (0.32 m) provided by the vehicles’ on-board sensors. Higher perception accuracy is also achieved in suburban areas.

**Figure 9:**
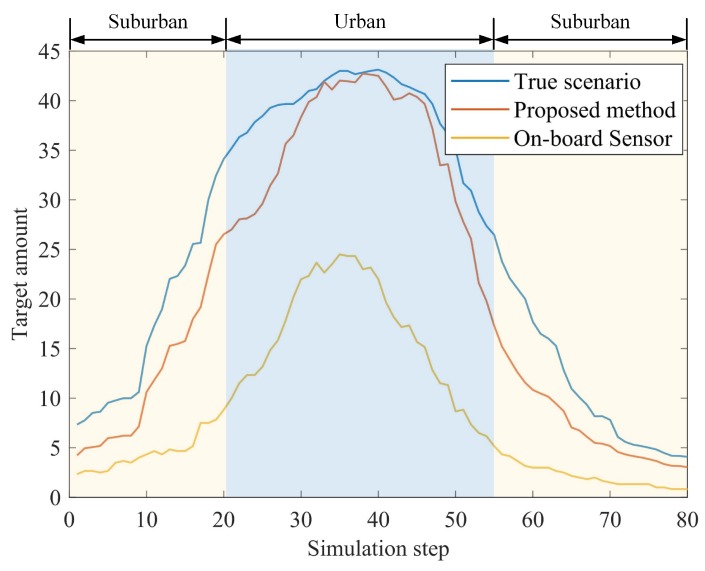
Perception integrity in terms of the number of targets captured.

Figure 9 shows the improvement in sensing integrity. The blue line is the true value of the number of targets within the demanding space. Based on the raw data of the on-board sensors, only 41.81% of targets are captured in the urban area, owing to occlusion or limited sensing range, while the proposed method enables 90.42% of the targets to be captured. The improvement comes from the sharing of information between the connected vehicles, and the information provided by the real-time dynamic map.

In summary, our approach significantly improves multiple-target positioning in terms of accuracy and integrity over that achieved using the original measurements, and is also more effective than other methods.

### 6.2. Adaptability to Different Scenarios

In the following section, we analyze the impact of different elements on the results of multiple-target positioning to demonstrate the adaptability of our method to different environments. Simultaneously, we discuss the contributions of different constraint types to the results.

#### 6.2.1. Number of Connected Vehicles

**Figure 10:**
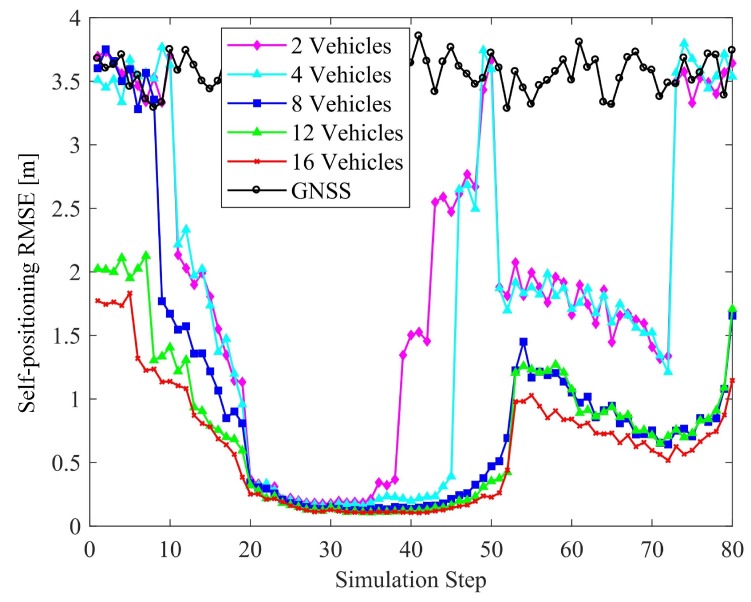
Effect of the number of connected vehicles on localization accuracy.

**Figure 11:**
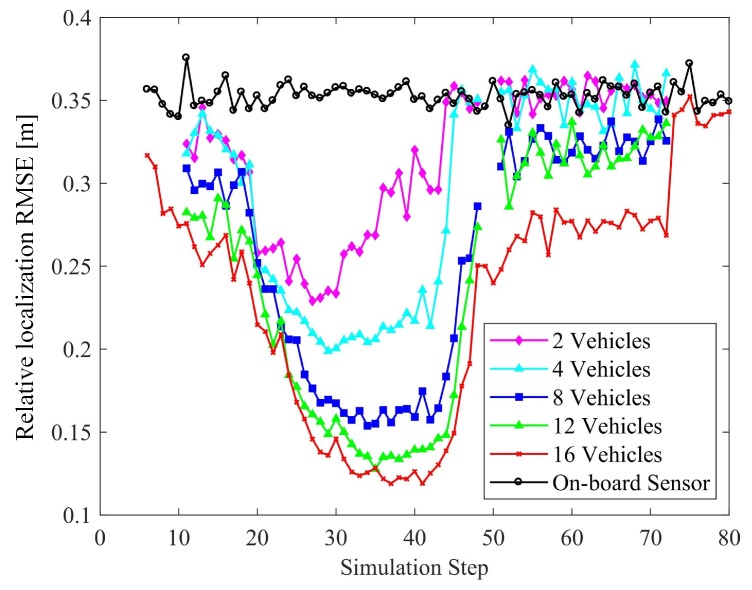
Effect of the number of connected vehicles on perception accuracy.

The vehicular positioning and relative localization of the targets in terms of the number of connected vehicles are shown in Figure 10 and Figure 11, respectively. Except for the number of connected vehicles, the configurations of the scene are identical, with 6 features and 3 objects. The accuracy of sensing and GNSS/INS are the same as those in previous experiments.

As can be seen from the figures, although there is a limited number of connected vehicles (only 2 vehicles), the positioning capacities of the vehicles and other targets are significantly improved in the urban areas. In general, the greater the number of connected vehicles, the smaller the corresponding positioning errors, as more V-V constraints are imposed between the vehicles. It is worth noting that there is a trend that the RMSEs of self-positioning increase as the simulation step increases from 70 to 80, as the vehicles are heading the end of the roads, where features and objects are becoming increasingly sparse. In theory, the lower bound of positioning error of 12 vehicles is lower than 8 vehicles. Due to the limited simulation times, the RMSE fluctuates near the theoretical bounds. Therefore, the RMSE of 12 vehicles seems close to those of 8 vehicles. However, based on the existing results, we cannot say that there is a trend that they will exceed those of 8 vehicles.

Another interesting observation is that for a given number of connected vehicles, the positioning errors in the suburban areas are larger than those in the urban areas, but it is worth noting that the decreasing trend of RMSE with increasing the number of connected vehicles is more significant. This is because, unlike the urban area with sufficient types of constraints, the constraints in suburbs are mainly of V-V. Therefore, we argue that the number of connected vehicles is important for improving location accuracy in the suburban area, which is consistent with the results shown in Figure 10.

As for the perception accuracy, in suburban area, with the increase of the number of vehicles, there are more constraints which benefits the positioning of connected vehicles and other objects. The underlying mechanism can be explained by Equation (36). As the constraints increase, the dimension of $h_{t}\left(\theta \right)$ increases, which leads to an increase in the value of the information matrix I(θ). The CRLB for $\theta_{m}$, which can be calculated with the diagonal element of ${I\left(\theta \right))}^{-1}$ will decrease, which leads to the reduction of the absolute positioning error for each object. As the perception result is gained by projecting the absolute of other targets to the vehicle-body coordinate system based on the self-positioning, the reduction of the absolute positioning error finally improves the perception accuracy.

#### 6.2.2. Number of Features

**Figure 12:**
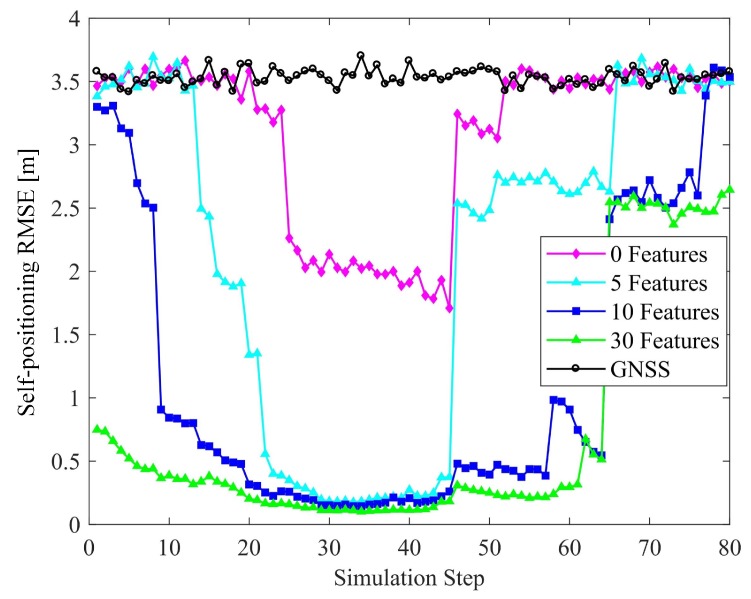
Effect of the number of features on localization accuracy.

**Figure 13:**
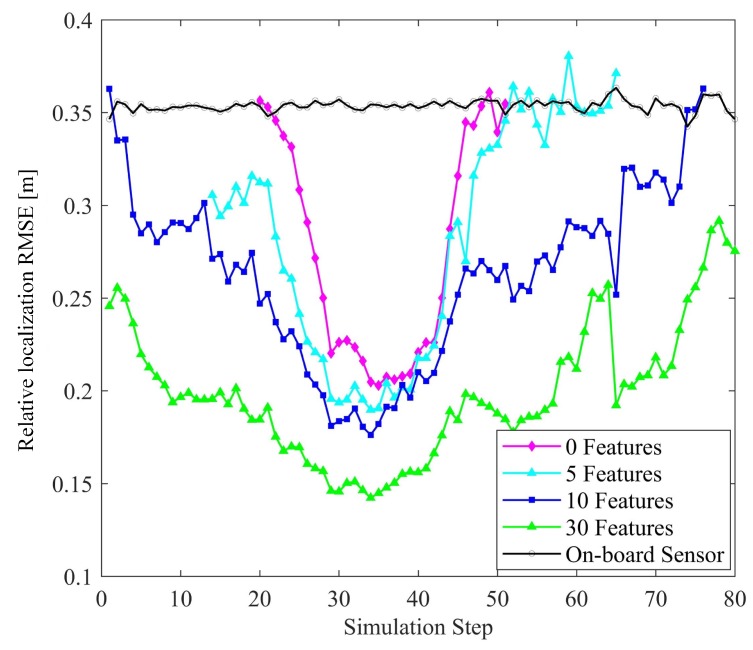
Effect of the number of features on perception accuracy.

The effect of the number of features are shown in Figure 12 and Figure 13. There are four connected vehicles running on the road with four objects and different numbers of features. It is obvious that increasing the number of features improves the accuracy of vehicle positioning and relative localization of the targets. Compared to raw measurements, even with a few features, the positioning and perception errors are reduced by exploiting the vehicle-to-target constraints and HD map information. It is noteworthy that at the intersection, the positioning accuracy is high when the number of features is 5, 10, or 30. However, if there is no feature i.e., no map information is used, the positioning error is obviously higher. This reflects the contribution of the HD map to vehicle positioning.

#### 6.2.3. Number of Objects

**Figure 14:**
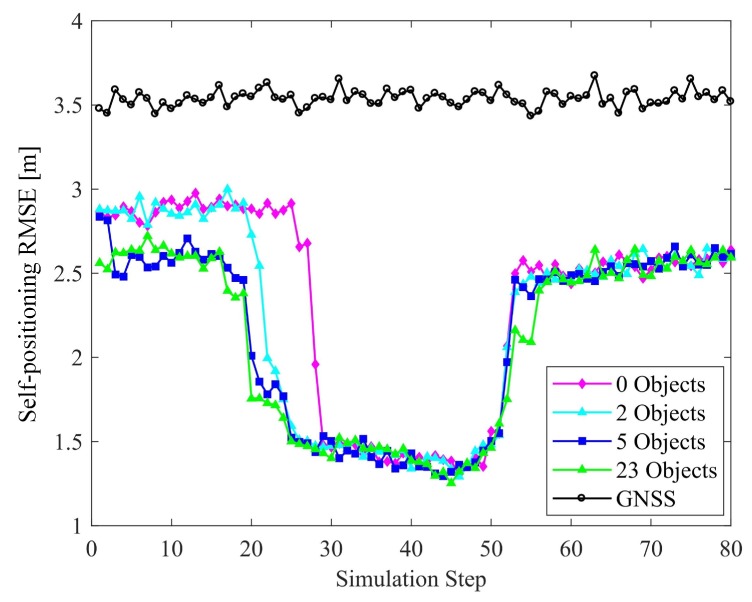
Effect of the number of objects on localization accuracy.

**Figure 15:**
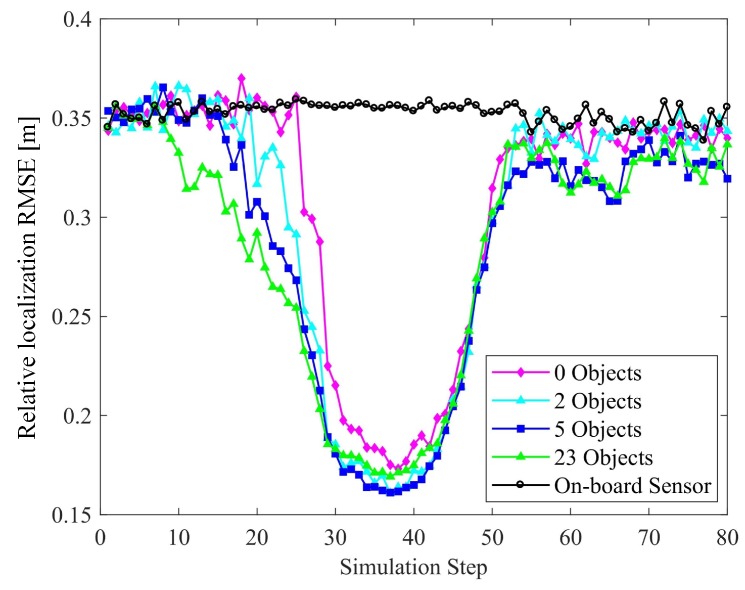
Effect of the number object on perception accuracy.

In comparing the results obtained when the number of objects varies, we set $N_{v}=8$ and $N_{f}=0$. The corresponding results are given in Figure 14 and Figure 15. In the suburban zones, the increase in the number of objects improves the positioning. However, it is noteworthy that such an improvement in the intersection zone is not obvious. The reason is that in the former zone, there are very few V-V constraints, and V-O constraints play the main role in improving the results. Hence, adding objects can effectively improve the positioning. However, at the intersections, the V-V constraints formed by 8 vehicles are dominant and the results approach the theoretical bounds achievable. There is no significant improvement in accuracy with the addition of objects.

In the case of relative localization, increasing the number of objects can reduce the overall perception accuracy, instead. As the number of objects increases, the proportion of objects among all the perceived targets participating in the perceptual precision calculation increases. The objects are less constrained relative to other targets, and the overall clarity of perception declines. Considering this and the former discussion, we argue that V-O constraints are less effective than V-V and V-F constraints in improving multiple-target positioning accuracy.

## 7. Conclusions

This study focuses on the problem of multiple-target positioning for ICVs. We propose a unified theoretical framework for positioning both vehicles and other targets, wherein sensor data from V2X and HD map data are effectively fused with GNSS/INS and on-board sensors. By jointly exploiting the vehicle-to-target constraints and HD map information, the vehicular localization accuracy can be enhanced to meet the requirements of high-level automated driving by using low-cost GNSS/INS and on-board sensors in urban areas. Meanwhile, the confidence and integrity of the results of relative localization of targets are significantly improved, realizing sensing beyond line of sight and field of view, which can improve the transportation efficiency and safety. Furthermore, the proposed framework is applicable to more challenging scenarios entailing fewer connected vehicles and sparse features and objects. In future research, we plan to remove the limiting assumption of data association employed in this study by applying association methods in the process of optimization. We will also study the formulation of communication delay of V2X in the data fusion framework.

## Figures and Tables

**Table 1:** Maps for different levels of Intelligent Connected Vehicles.

**Grade**	**Title**	**Map**	**Accuracy**	**Typical** **Condition**
Driver Scenario				
**1 (DA)**	Driver Assistance	ADAS	Submeter	Optional
**2 (PA)**	Partial Autopilot	ADAS	Submeter	Optional
Automatic Driving Sys. Scenario		ADAS + HD	Submeter Centimeter	Optional
**3 (CA)**	Conditional Autopilot			
**4 (HA)**	High-Level Automated Driving	ADAS + HD	Submeter Centimeter	Essential
**5 (FA)**	Completely Automated Driving	HD	Centimeter	Essential (auto updated)
